# l-Isoleucine Administration Alleviates Rotavirus Infection and Immune Response in the Weaned Piglet Model

**DOI:** 10.3389/fimmu.2018.01654

**Published:** 2018-07-16

**Authors:** Xiangbing Mao, Changsong Gu, Man Ren, Daiwen Chen, Bing Yu, Jun He, Jie Yu, Ping Zheng, Junqiu Luo, Yuheng Luo, Jianping Wang, Gang Tian, Qing Yang

**Affiliations:** ^1^Animal Nutrition Institute, Sichuan Agricultural University, Chengdu, China; ^2^Key Laboratory of Animal Disease-Resistance Nutrition, Chinese Ministry of Education, Chengdu, China; ^3^College of Animal Science, Anhui Science and Technology University, Fengyang, China; ^4^Department of Animal Science, Oklahoma State University, Stillwater, OK, United States

**Keywords:** isoleucine, rotavirus, immune response, pattern recognition receptors, weaned piglet

## Abstract

Rotavirus (RV) infection is one of the main pathogenic causes of severe gastroenteritis and diarrhea in infants and young animals. This study aimed to determine how dietary l-isoleucine supplementation improves the growth performance and immune response in weaned piglets with RV infection. In cell culture experiment, after IPEC-J2 and 3D4/31 cells were treated by 8 mM l-isoleucine for 24 h, the gene expressions of β-defensins and pattern recognition receptors (PRR) signaling pathway were significantly increased. Then, in the *in vivo* experiment, 28 crossbred weaned pigs were randomly divided into two groups fed with basal diet with or without l-isoleucine for 18 days. On the 15th day, the oral RV gavage was executed in the half of piglets. Average daily feed intake and gain of piglets were impaired by RV infection (*P* < 0.05). RV infection also induced severe diarrhea and the increasing serum urea nitrogen concentration (*P* < 0.05), and decreased CD4^+^ lymphocyte and CD4^+^/CD8^+^ ratio of peripheral blood (*P* < 0.05). However, dietary l-isoleucine supplementation attenuated diarrhea and decreasing growth performance (*P* < 0.05), decreased the NSP4 concentration in ileal mucosa, and enhanced the productions and/or expressions of immunoglobulins, RV antibody, cytokines, and β-defensins in serum, ileum, and/or mesenteric lymph nodes of weaned piglets (*P* < 0.05), which could be relative with activation of PRR signaling pathway and the related signaling pathway (*P* < 0.05) in the weaned pigs orally infused by RV. These results indicate that dietary l-isoleucine could improve the growth performance and immune function, which could be derived from l-isoleucine treatment improving the innate and adaptive immune responses *via* activation of PRR signaling pathway in RV-infected piglets. It is possible that l-isoleucine can be used in the therapy of RV infection in infants and young animals.

## Introduction

Rotavirus (RV), a double-strained RNA (dsRNA) virus of the family *Reoviridae*, is the main cause of severe diarrhea hospitalization among infants, and causing ~527,000 deaths per year worldwide (especially developing country) (1–3). The previous study revealed that the replication of RV was restricted in the interferon (IFN)-treated cells (4), which indicates that IFN may play a vital role in antiviral immune response. RV replication is recognized by pattern recognition receptors (PRRs), such as toll-like receptor 3 (TLR3), retinoic acid inducible gene I (RIG-I), and/or melanoma differentiation-associated protein 5 (MDA5), and then activates the interferon regulatory factor 3 (IRF3) and nuclear factor-kappa B (NF-κB) that lead to the expression of type I IFN and pro-inflammation cytokines (5, 6). However, during the initial stage of RV infection, RV may suppress the expression of type I IFN and pro-inflammation cytokines through stimulating the proteasome-mediated degradation of IRF3 and NF-κB (4, 7, 8). These may impair the antiviral immune response and lead to a severe gastroenteritis.

Isoleucine is a kind of branched chain amino acid, which is essential for some physiological functions of humans and pigs (7, 9–11). Burns (9) has shown that isoleucine will specifically be incorporated into proteins of immune cells, such as lymphocytes, eosinophils, and neutrophils. And, in humans and animals, isoleucine plays a critical role for immune functions, including maintaining the development of immune organs and cells, and stimulating the secretion of immune molecules substances (12–16). l-Isoleucine administration can improve the health status of cells and animals under some challenge conditions (17, 18). In recent clinical study, dietary l-isoleucine supplementation can relieve the acute diarrhea induced by the malnourishment in children, which is related to the production of host defense peptides induced by isoleucine (19). Thus, isoleucine has the capacity of preventing the invasion of pathogens *via* the increase of immunity.

The aim of this study was to verify the hypothesis that supplementing l-isoleucine in diets might alleviate the effect of RV infection on growth and diarrhea in piglets, and analyze the possible change and mechanism of immune response during this process. We wanted to research the effect of isoleucine on gut. In the gut, there are including many types of cells, such as epithelial cells and immune cells. IPEC-J2 is a kind of porcine intestinal epithelial cell line. However, we chose to culture alveolar macrophage (3D4/31) that is a kind of porcine immune cell line in the cell culture experiment, which was used to evaluate the effect of l-isoleucine on immune cell. Previous studies have shown that there is the resemblance between humans and pigs (including gut function and immunity) (20), so pigs are usually considered as a research model for humans. Moreover, piglets are susceptible to heterotypic RV infection that leads to the serious diarrhea (21), which is similar to infants and young children. Thus, piglets are an ideal animal model on studying the effect of RV infection on immune response *in vivo*.

## Materials and Methods

### Cell Culture Experiment

#### Materials

Dulbecco’s Modified Eagle’s Medium/F12 (DMEM/F12) medium, Roswell Park Memorial Institute (RPMI) 1640 medium, and insulin–transferrin–selenium (ITS) solution were purchased from Lonza (Walkersville, MD, USA). Antibiotics (penicillin–streptomycin solution), trypsin–EDTA solution, and fetal bovine serum (FBS) were from Atlanta Biologicals (Flowery Branch, GA, USA). Sodium pyruvate solution was purchased from Hyclone Laboratories Inc. (Logan, UT, USA). RNAzol RT was from Molecular Research Center (Cincinnati, OH, USA). The iScript™ complementary DNA (cDNA) Synthesis Kit and iTaq™ Universal SYBR Green Supermix were purchased from Bio-Rad Laboratories, Inc. (Hercules, CA, USA). The genes included TLR3, retinoic-acid-inducible protein 1 (RIG-I), IRF3, mitochondrial antiviral-signaling protein (MAVS), MDA5, NF-κB, interferon beta (IFNβ), IFNγ, porcine beta-defensin 2 (pBD2), and pBD3. The primers (Table S1 in Supplementary Material) were from Integrated DNA Technologies, Inc. (Coralville, IA, USA).

#### Cell Culture

The IPEC-J2 cell line (porcine intestinal epithelial cells) and 3D4/31 cell line (porcine lung alveolar macrophage cells) were kindly provided by Dr. Guolong Zhang at Oklahoma State University (Stillwater, OK, USA). Both cells were cultured as described previously with some modifications (22). Briefly, IPEC-J2 cells were cultured in 6-well plates at 2.5 × 10^5^ cells/well with DMEM/F12 medium added 10% FBS, 1% antibiotics, and 0.2% ITS. 3D4/31 cells were cultured in 6-well plates at 2.5 × 10^5^ cells/well with RPMI 1640 medium added 10% FBS, 1% antibiotics, and 1 mM sodium pyruvate. According to some previous studies, different doses (0.4–10 mM) of isoleucine can affect the physiological functions in different cells (10, 23–25). Combining with our pre-experiments, we found that 8 mM of isoleucine can regulate the mRNA expressions of some genes (data no published). Thus, 8 mM was used as the treating dose in this experiment. Following 16–20 h culture, cells were treated by 8 mM l-isoleucine for 24 h (*n* = 6).

#### RNA Isolation and Quantitative Real-Time PCR

The RNA isolation and quantitative real-time PCR were performed by described previously with some modifications (22). Following l-isoleucine treatment, the RNA of cells was extracted by using RNAzol according to the manufacturer’s instruction. The RNA concentrations of all samples were analyzed by Nanodrop (Thermo Scientific, Wilmington, DE, USA). The first strand of cDNA was synthesized with iScript™ cDNA Synthesis Kit according to the manufacturer’s instruction. And then, in MyiQ Real-Time PCR System (Bio-Rad), quantitative real-time PCR was performed with iTaq™ Universal SYBR Green Supermix as described previously. Cycling conditions were as follows: 95°C for 30 s, and 40 cycles involving a combination of 94°C for 5 s and 60°C for 30 s. The gene-specific primers used in this study are listed in Table S1 in Supplementary Material (22, 26). Relative gene expression to the reference gene (β-actin) was performed and calculated according to the ΔΔCt method as described previously (22).

### Animal Experiment

#### Animals, Diets, and Experimental Design

The experimental protocol was approved by Animal Care Advisory Committee of Sichuan Agricultural University. All the test operations were executed with documents to Regulations on Animal Welfare and Animal Testing and carried out at the Experimental Farm of Sichuan Agricultural University. The animal Ethics Committee approval number is CD-SYXK-2017-015. Twenty-eight DLY (Duroc × Landrace × Yorkshire) weaned barrows (6.95 ± 0.41 kg) were fed in the metabolic cages (1.5 m × 0.7 m × 1.0 m) with a self-feeder and a nipple watering device, which were located in a temperature-controlled room.

The basal diet was formulated to meet National Research Council-recommended nutrient requirements (27) for pigs (7–11 kg) (28). And all the diets were prepared at the Experimental Farm of Sichuan Agricultural University. The composition and nutrient content of basal diet were presented in Table 1. Either 1% (w/w) l-isoleucine or 0.68% (w/w) l-alanine (isonitrogenous control) was added to the basal diet through replacing the corn starch.

**Table 1:** Composition of the basal diets used in this study (as-fed basis).

Ingredients	g/kg
Corn	619
Soybean meal	60
Soy protein concentrate	100
Fish meal	50
Whey powder	50
Soybean oil	30
Corn starch	33
Glucose	20
CaCO_3_	7
CaHPO_4_	10
Salt	3
Chloride choline	1.5
Vitamin premix^a^	0.3
Mineral premix^b^	3
l-Lys·HCl	4.3
dl-Met	0.6
l-Thr	0.8
l-Trp	0.4
l-Val	0.3
l-Ala	6.8
Total	1,000
Energy and nutrition levels (%)^c^	
Digestible energy (MJ/kg)	14.05
Crude protein	19.79
Ca	0.8
Available P	0.48
Digestible Lys	1.35
Digestible Met	0.39

*^a^Vitamin premix provided the following (per kg of diet): vitamin A, 5,512 IU; vitamin D_3_, 200 IU; vitamin E, 24 mg; vitamin K_3_, 3 mg; vitamin B_1_, 3 mg; vitamin B_2_, 6 mg; vitamin B_6_, 3 mg; vitamin B_12_, 24 µg; d-pantothenic acid, 15 mg; nicotinic acid, 30.3 mg; folic acid, 1.2 mg; biotin, 150 µg*.

*^b^Mineral premix provided the following (per kg of diet): Fe, 60 mg; Cu, 4 mg; Zn, 60 mg; Mn, 2 mg; Se, 0.10 mg; I, 0.14 mg*.

*^c^Nutrient levels represent the calculated values*.

Rotavirus used in this study was a tissue culture-adapted Ohio State University strain (ATCC#VR-893). RV preparation and virus titer determination [tissue culture infective dose 50 (TCID_50_)] were determined as described previously (28, 29).

Following 3 days for orientation, piglets were divided into two groups on basis of body weight and litter origin and were fed with basal diet with 0.68% l-alanine (CON group, *n* = 14) or with 1% l-isoleucine (Ile group, *n* = 14) for 18 days. On 15th day, all piglets were infused 5 mL of sterile NaHCO_3_ solution (100 mmol/L). Then, half of piglets in two groups were orally administrated with 4 mL (10^6^ TCID_50_/mL) of RV while the others were infused with 4 mL of the sterile medium. After RV treatment, the diarrhea state was recorded in each day. Fecal consistency was scored as follows: 0, normal; 1, pasty; 2, semiliquid; and 3, liquid. Piglets were considered as diarrhea when fecal consistency score is ≥2 (30). Average daily gain (ADG), average daily feed intake (ADFI), feed/gain ratio (F/G ratio), and gain (G) were calculated *via* weighing body weight and feed intake of all piglets at 08:00 of days 1, 15, and 19.

#### Tissue Sample Collection

The blood of pigs were gathered through the jugular vein, and 5 mL of whole blood was added in EDTA-coated tube for lymphocyte subtypes assay, then the residual blood was used to separate serum sample by centrifugation at 3,500 *g* for 15 min. The isolated serum samples were stored at −20°C until analysis. Following sample collection, all piglets were euthanized *via* intracardially injecting Na pentobarbital (50 mg/kg of body weight) and exsanguinated. The abdomen was immediately opened. The mucosa of mid ileum and mesenteric lymph nodes were removed, flushed with ice-cold saline solution, frozen in liquid nitrogen, and then stored at −80°C until analysis.

#### Serum Urea Nitrogen (UN) and Amino Acid Assay

Serum UN concentration was analyzed with an assay kit from Nanjing Jiancheng Biochemistry Institute (Nanjing, China) according to the manufacturer’s instructions. Serum-free amino acid concentrations were determined by ion-exchange chromatography with physiological fluid analysis conditions (L-8900 AA Analyzer, Hitachi, Japan) as described previously (29).

#### Lymphocyte Subtype Assay

Premixed cocktail of monoclonal antibodies: CD3-PerCP, CD4-FITC, and CD8-PE (BD Biosciences, USA) were added into 100 µL of EDTA blood in a 12 mm × 75 mm tube. The tube was gently mixed and incubated at room temperature for 20 min in the dark, then added 2 mL of RBC lysing solution (BD Biosciences, USA) and incubated for another 10 min. Centrifugation at 500 *g* for 5 min and subsequently washed with 2 mL of PBS, the cell pellets were resuspended in 0.5 mL of 1% paraformaldehyde in PBS. The stained cells were then analyzed by a flow cytometer (FACSverse, BD Biosciences, USA), using CellQuest software program (BD Biosciences, USA). The percentage of CD3^+^, CD4^+^, and CD8^+^ lymphocytes and CD4^+^/CD8^+^ were determined from the CD3^+^/CD4^+^ and CD3^+^/CD8^+^ tubes, using FL1 and FL2 (31).

#### Serum and Ileal Cytokines Assay

Eighty mg of ileum was added into ice-cold PBS solution and executed the pulverization by using ultrasonic cell disruption system (Scientz-IID, Scientz, Ningbo, China) at 4°C, and then centrifuged at 3,500 *g* for 15 min at 4°C. The ileal supernatant and serum was used to detect the IFNβ, IFNγ, interleukin 1β (IL-1β), IL-10, and tumor necrosis factor α (TNF-α) concentrations with commercially available porcine enzyme-linked immunosorbent assay (ELISA) kits (Xinle Bio Co. Ltd., Shanghai, China) according to the manufacturer’s instructions.

#### Serum and Ileal NSP4, RV Antibody (RV-ab), and Immunoglobulin Assay

The concentration of RV-ab and NSP4 (non-structural protein) in serum and ileum were detected with a commercially available porcine ELISA kit (TSZ, Framingham, MA, USA) according to the manufacturer’s instructions. The concentrations of immunoglobulins (IgA, IgM, and IgG) in serum and secretory IgA (sIgA) in ileum were determined with available porcine ELISA kits (Xinle Bio Co. Ltd., Shanghai, China) according to the manufacturer’s instructions.

#### Total RNA Isolation and Real-Time Quantitative PCR

The materials and methods of total RNA isolation and quantitative real-time PCR are the same as the cell culture experiment. The genes included TLR3, RIG-I, MDA5, MAVS, toll-like receptor-associated activator of interferon (TRIF), TNF receptor associated factor 3 (TRAF3), NF-κB essential modulator (NEMO), transforming growth factor-β-activating kinase 1 (TAK1), IRF3, NF-κB, IL-1β, IL-10, TNF-α, IFNβ, IFNγ, PG1–5 pBD1, pBD2, and pBD3. The gene-specific primers used in this study are listed in Table S1 in Supplementary Material (22, 26). Relative gene expression to the reference gene (β-actin) was performed and calculated according to the ΔΔCt method as described previously (22).

## Statistics Analysis

### Cell Culture Experiment

In cell experiments, statistical analyses were performed with GraphPad PRISM 5 (GraphPad Software, La Jolla, CA, USA). Data were analyzed using unpaired Student’s two-tailed *t* test. Statistical significance was considered at *P* < 0.05.

### Animal Experiment

In animal experiment, the growth performance data of all piglets before RV infusion were analyzed with the unpaired *t* test. The diarrhea rate data of all piglets were analyzed with chi-square test. The other data were analyzed as a 2 × 2 factorial with the general linear model procedures of the Statistical Analysis Package. The model factors included the effects of l-isoleucine administration, RV infection and their interaction. All data were analyzed with SPSS (Version 21.0; IBM, USA) and indicated as means with their SEs. *P* < 0.05 was considered statistical significance while *P* < 0.10 was considered statistical tendency.

## Results

### Cell Culture Experiment

#### l-Isoleucine Regulates the Expressions of Innate Immunity-Relative Genes in IPEC-J2 and 3D4/31 Cells

**Figure 1:**
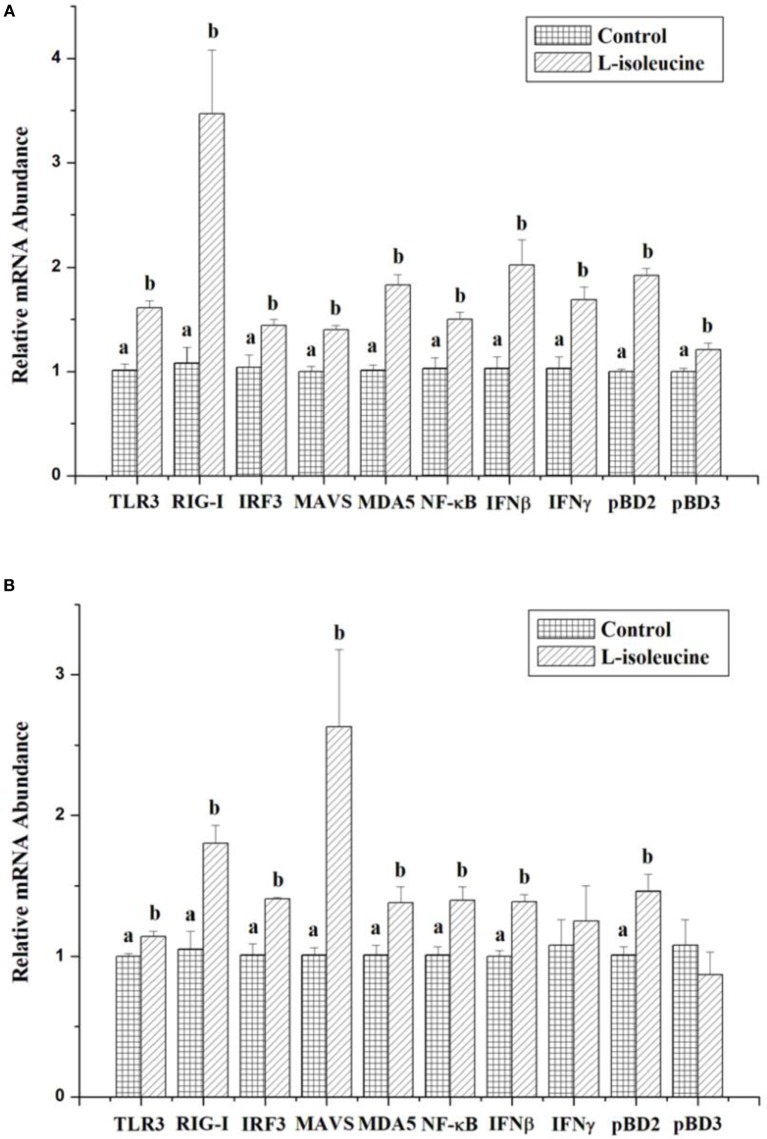
The effect of l-isoleucine treatment on the expressions of innate immunity-relative genes in IPEC-J2 **(A)** and 3D4/31 **(B)** cells. The relative mRNA abundance for the innate immunity-relative genes was normalized to that for β-actin. Values are means ± SEM; *n* = 6. Values with different letters are significantly different (*P* < 0.05).

Recent studies have shown that isoleucine treatment can improve the immune functions of cells, animals and humans under normal and disease conditions (10, 14, 18, 19). To initially understand the effect of isoleucine on innate immunity, we measured the expressions of innate immunity-relative genes after isoleucine treatment in IPEC-J2 and 3D4/31 cells. And in this study, 8 mM l-isoleucine treatment significantly increased the expressions of TLR3, RIG-I, IRF3, MAVS, MDA5, NF-κB, IFNβ, IFNγ, pBD2, and pBD3 in IPEC-J2 cells (*P* < 0.05, Figure 1A) and significantly enhanced the expressions of TLR3, RIG-I, IRF3, MAVS, MDA5, NF-κB, IFNβ, and pBD2 in 3D4/31 cells (*P* < 0.05, Figure 1B).

### Animal Experiment

#### Growth Performance

During entire trial period, there were not any unexpected deaths. Due to the RV infection, some piglets showed weight losing, and then G were calculated and analyzed instead of ADG and F/G ratio during 15–18 days. Dietary l-isoleucine supplementation increased ADG and ADFI and reduced F/G ratio of piglets in the first 14 days of trial (*P* < 0.05, Table 2). RV infusion reduced ADFI and G of weaned piglets (*P* < 0.05). But dietary l-isoleucine supplementation attenuated the effect of RV infection on ADFI and G in weaned piglets (*P* < 0.05).

**Table 2:** The effect of dietary l-isoleucine supplementation and/or rotavirus (RV) challenge on the growth performance in the weaned pig.

	RV−		RV+		*P*-value		
	CON	Ile	CON	Ile	Ile	RV	Ile × RV
**1–14 days (*n* = 14)**							
ADFI (g)	227.61 ± 14.37	285.10 ± 17.27			<0.05		
ADG (g)	150.12 ± 10.01	210.34 ± 14.02			<0.05		
F/G	1.60 ± 0.09	1.32 ± 0.09			<0.05		

**15–18 days (*n* = 7)**							
ADFI (g)	388.33 ± 26.46^B^	454.17 ± 22.91^A^	272.78 ± 26.46^C^	409.00 ± 20.50^A^	<0.05	<0.05	0.17
G (kg)	0.90 ± 0.22^A^	0.96 ± 0.13^A^	-0.51 ± 0.12^B^	0.75 ± 0.22^A^	<0.05	<0.05	0.05

*RV−, infusing the essential medium; RV+, infusing the porcine rotavirus; CON, l-alanine-supplemented diet; Ile, l-isoleucine-supplemented diet*.

*^A,B,C^In the same row, values with different letter superscripts mean significant difference (*P* < 0.05)*.

#### Serum UN and Free AA Concentrations

Rotavirus infusion increased serum UN (*P* < 0.05), proline (*P* < 0.05), cysteine (*P* < 0.05), arginine (*P* < 0.05), serine (*P* = 0.05), glycine (*P* = 0.09), and alanine (*P* = 0.08) concentrations in weaned piglets (Table 3). Dietary l-isoleucine supplementation decreased serum UN (*P* < 0.05) and glycine (*P* = 0.06) concentrations, increased serum isoleucine (*P* < 0.05) and arginine (*P* = 0.06) concentrations, and attenuated the effect of RV infection on serum UN and glycine concentrations in weaned piglets (*P* < 0.05, Table 3).

**Table 3:** The effect of dietary l-isoleucine supplementation and/or rotavirus (RV) challenge on the concentrations of amino acids and nitrogen in the serum of weaned pigs (*n* = 7).

	RV−		RV+		*P*-value		
	CON	Ile	CON	Ile	Ile	RV	Ile × RV
**Free AA (μM)**							
Asp	30.62 ± 2.62	29.84 ± 2.62	32.02 ± 2.62	31.91 ± 2.62	0.14	0.47	0.37
Thr	38.12 ± 4.76	34.54 ± 4.76	38.12 ± 4.76	29.78 ± 4.76	0.19	0.20	0.05
Ser	58.85 ± 4.20^BC^	54.29 ± 4.20^C^	69.36 ± 3.15^A^	62.00 ± 4.20^AB^	0.45	0.05	0.54
Glu	254.53 ± 19.13	256.01 ± 19.13	232.47 ± 19.13	260.42 ± 19.13	0.24	0.76	0.54
Gly	380.60 ± 31.53^AB^	334.27 ± 31.53^B^	410.19 ± 31.53^A^	351.33 ± 34.53^B^	0.06	0.09	0.22
Ala	198.67 ± 14.25	201.34 ± 13.36	219.16 ± 16.04	221.83 ± 14.25	0.74	0.08	0.99
Val	97.23 ± 7.03	91.46 ± 7.03	90.83 ± 7.03	96.15 ± 7.03	0.83	0.96	0.53
Cys	3.83 ± 1.21^B^	3.63 ± 1.21^B^	6.06 ± 1.21^A^	4.85 ± 1.21^AB^	0.33	<0.05	0.53
Met	23.87 ± 2.98	22.38 ± 2.98	26.86 ± 2.98	22.38 ± 2.98	0.24	0.67	0.40
Ile	39.50 ± 3.94^B^	63.35 ± 3.94^A^	43.52 ± 3.94^B^	68.54 ± 2.62^A^	<0.05	0.47	0.56
Leu	86.57 ± 6.56	87.88 ± 6.56	90.51 ± 6.56	95.75 ± 6.56	0.85	0.20	0.93
Tyr	61.60 ± 3.62	58.92 ± 3.62	59.79 ± 3.62	57.98 ± 3.62	0.12	0.16	0.22
Phe	59.47 ± 6.61	57.82 ± 6.61	59.47 ± 6.61	64.42 ± 6.61	0.92	0.16	0.54
Trp	20.42 ± 4.08	26.55 ± 4.08	22.47 ± 4.08	26.55 ± 4.08	0.61	0.27	0.26
Lys	114.03 ± 20.47	127.19 ± 17.54	135.96 ± 16.08	133.03 ± 16.08	0.72	0.13	0.64
His	48.05 ± 4.65	48.85 ± 3.10	48.05 ± 3.10	54.25 ± 3.10	0.40	0.15	0.30
Arg	67.94 ± 12.19^C^	81.87 ± 10.45^B^	99.29 ± 10.45^A^	102.78 ± 10.45^A^	0.06	<0.05	0.78
Pro	81.74 ± 8.06^B^	89.80 ± 6.91^AB^	99.01 ± 6.91^AB^	110.52 ± 6.91^A^	0.15	<0.05	0.57
UN (mM)	4.32 ± 1.12^B^	3.30 ± 1.05^B^	9.46 ± 1.39^A^	4.02 ± 1.12^B^	<0.05	<0.05	0.13

*RV−, infusing the essential medium; RV+, infusing the porcine rotavirus; CON, l-alanine-supplemented diet; Ile, l-isoleucine-supplemented diet*.

*^A,B,C^In the same row, values with different letter superscripts mean significant difference (*P* < 0.05)*.

#### Diarrhea Status, RV-ab, and NSP4 Concentration of Serum and Ileum

**Figure 2:**
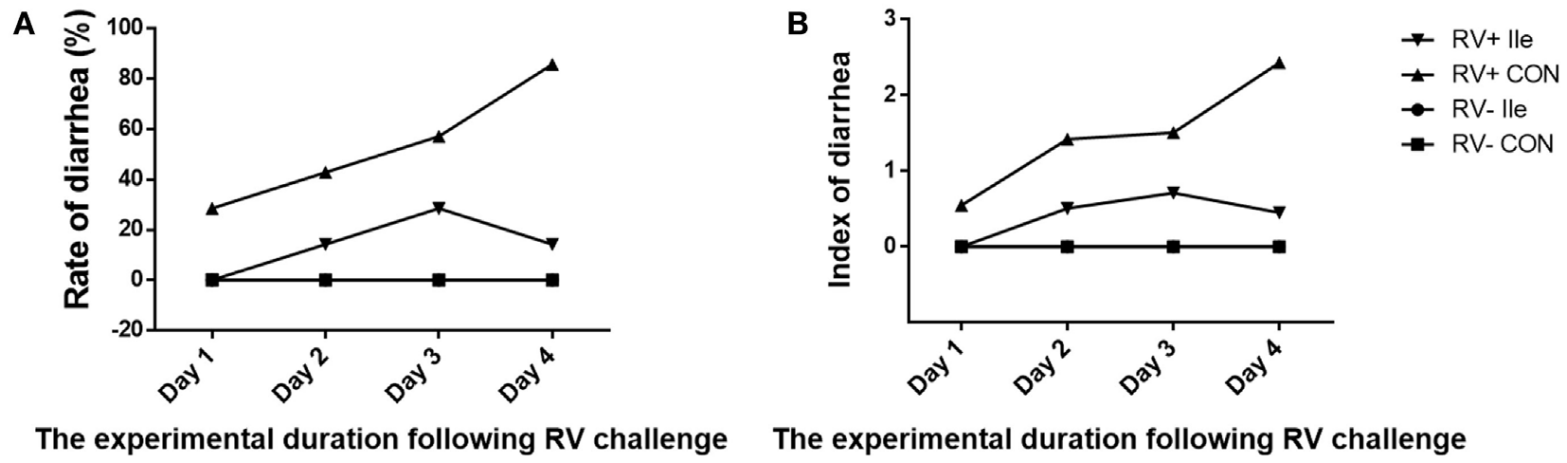
Dynamic variation of diarrhea rate **(A)** and index **(B)** of weaned piglets by adding l-Ile after RV challenged over time.

Following RV challenge, diarrhea rate was increased (Figure 2; Table 4), serum and ileal RV-ab and NSP4 concentrations were enhanced (*P* < 0.05, Table 5) in weaned piglets. Furthermore, in the weaned piglets challenged by RV, l-isoleucine administration attenuated the increasing NSP4 level (*P* < 0.05, Table 5), diarrhea rate, shortened the duration of diarrhea (Figure 2; Table 4), but further enhanced serum and ileal RV-Ab levels (*P* < 0.05, Table 5).

**Table 4:** The effect of dietary Ile supplementation and/or RV challenge on the diarrhea rate in weaned pigs.

Treat	*N*	Normal	Diarrhea	Diarrhea rate %	*P*_Ile_	*P*_RV_
CON	7	7	0	0	<0.05	<0.05
Ile	7	7	0	0		
CON + RV	7	1	6	85.7		
Ile + RV	7	5	2	28.5		

*RV, infusing the porcine rotavirus; CON, l-alanine-supplemented diet; Ile, l-isoleucine-supplemented diet*.

**Table 5:** The effect of dietary l-isoleucine supplementation and/or rotavirus (RV) challenge on the contents of RV antibody (RV-ab) and NSP4 in the ileum and/or serum of weaned pigs (*n* = 7).

	RV−		RV+		*P*-value		
	CON	Ile	CON	Ile	Ile	RV	Ile × RV
**RV-ab**							
Serum (μg/mL)	5.37 ± 0.34^C^	5.64 ± 0.20^C^	9.13 ± 0.34^B^	12.71 ± 0.19^A^	<0.05	<0.05	<0.05
Ileum (μg/mg prot)	0.98 ± 0.06^C^	1.07 ± 0.05^C^	2.23 ± 0.07^B^	2.68 ± 0.06^A^	<0.05	<0.05	0.21

**NSP4**							
Ileum (ng/mg prot)	26.91 ± 3.31^C^	23.66 ± 3.31^C^	130.35 ± 3.63^A^	77.14 ± 3.31^B^	<0.05	<0.05	<0.05

*RV−, infusing the essential medium; RV+, infusing the porcine rotavirus; CON, l-alanine-supplemented diet; Ile, l-isoleucine-supplemented diet*.

*^A,B,C^In the same row, values with different letter superscripts mean significant difference (*P* < 0.05)*.

#### Lymphocyte Subpopulation

In Table 6, the effect of RV challenge and l-isoleucine administration on lymphocyte subpopulation in the peripheral blood of weaned piglets was shown. RV infusion decreased CD4^+^ rate and CD4^+^/CD8^+^ ratio in the peripheral blood (*P* < 0.05). However, dietary l-isoleucine supplementation attenuated the effect of RV infection on CD4^+^ rate in the peripheral blood (*P* < 0.05).

**Table 6:** The effect of dietary l-isoleucine supplementation and/or rotavirus (RV) challenge on the subgroup of lymphocytes in the serum in weaned pigs (*n* = 7).

	RV−		RV+		*P*-value		
	CON	Ile	CON	Ile	Ile	RV	Ile × RV
CD3^+^ (%)	66.82 ± 2.43	65.44 ± 2.43	63.33 ± 2.22	65.33 ± 2.22	0.89	0.45	0.48
CD4^+^ (%)	30.03 ± 1.42^A^	28.98 ± 1.17^A^	24.23 ± 1.74^B^	27.40 ± 1.31^A^	0.06	<0.05	0.27
CD8^+^ (%)	16.20 ± 1.07	17.63 ± 0.97	17.25 ± 0.97	17.46 ± 1.07	0.52	0.45	0.35
CD4^+^/CD8^+^	1.78 ± 0.11^A^	1.69 ± 0.12^AB^	1.49 ± 0.10^B^	1.57 ± 0.10^AB^	0.73	<0.05	0.26

*RV−, infusing the essential medium; RV+, infusing the porcine rotavirus; CON, l-alanine-supplemented diet; Ile, l-isoleucine-supplemented diet*.

*^A,B,C^In the same row, values with different letter superscripts mean significant difference (*P* < 0.05)*.

#### Serum and Ileal Concentrations of Immunoglobulins and Cytokines

Rotavirus infusion increased the serum IgA, IgG, IFNβ, IFNγ, IL-1β, and TNF-α levels in weaned piglets (*P* < 0.05, Table 7). However, dietary l-isoleucine supplementation also enhanced the serum IgA, IgG, IFNβ, IFNγ, IL-1β, TNF-α, and IL-10 levels and further promoted serum concentrations of these immunoglobulins and cytokines in the weaned piglets infected by RV (*P* < 0.05, Table 7).

**Table 7:** The effect of dietary l-isoleucine supplementation and/or rotavirus (RV) challenge on immunoglobulin and cytokine levels in the serum and ileum of weaned pigs (*n* = 7).

	RV−		RV+		*P*-value		
	CON	Ile	CON	Ile	Ile	RV	Ile × RV
**Serum**							
IgA (μg/mL)	94.16 ± 11.23^C^	128.73 ± 13.56^BC^	150.27 ± 13.56^B^	200.84 ± 13.56^A^	<0.05	<0.05	0.65
IgM (mg/mL)	2.26 ± 0.37	3.361 ± 0.49	2.97 ± 0.30	2.41 ± 0.42	0.26	0.45	0.86
IgG (mg/mL)	11.07 ± 1.07^C^	14.45 ± 1.07^B^	15.20 ± 0.96^B^	19.37 ± 1.07^A^	<0.05	<0.05	0.71
IFNβ (ng/L)	518.18 ± 23.29^C^	638.15 ± 21.26^B^	651.95 ± 23.29^B^	721.96 ± 21.26^A^	<0.05	<0.05	0.23
IFNγ (ng/L)	185.71 ± 17.96^C^	194.03 ± 13.58^BC^	233.35 ± 16.07^B^	283.32 ± 16.06^A^	<0.05	<0.05	0.21
IL-1β (ng/L)	12.83 ± 0.64^C^	16.10 ± 0.64^B^	17.07 ± 0.70^B^	19.47 ± 0.71^A^	<0.05	<0.05	0.57
TNF-α (ng/L)	95.03 ± 6.44^C^	113.90 ± 7.20^BC^	120.67 ± 8.74^B^	147.28 ± 6.99^A^	<0.05	<0.05	0.67
IL-10 (pg/mL)	117.90 ± 7.70^C^	132.97 ± 7.16^B^	119.56 ± 7.70^BC^	166.88 ± 7.29^A^	<0.05	0.15	0.13

**Ileum**							
IFNβ (pg/mg prot)	39.60 ± 2.58^C^	58.07 ± 3.08^B^	61.63 ± 2.58^B^	84.81 ± 3.08^A^	<0.05	<0.05	<0.05
IFNγ (pg/mg prot)	45.26 ± 3.06^C^	62.29 ± 3.42^AB^	59.70 ± 2.79^B^	69.40 ± 3.06^A^	<0.05	<0.05	0.58
IL-1β (ng/mg prot)	3.53 ± 0.21^B^	3.84 ± 0.24^B^	3.92 ± 0.27^AB^	4.34 ± 0.27^A^	0.07	0.09	0.48
IL-10 (pg/mg prot)	39.96 ± 2.13^AB^	42.02 ± 2.13^AB^	36.68 ± 2.12^B^	45.69 ± 1.94^A^	<0.05	0.14	0.21
SIgA (μg/mg prot)	12.82 ± 0.84^C^	14.09 ± 0.79^B^	12.86 ± 0.72^BC^	16.93 ± 0.71^A^	<0.05	0.09	0.11
TNF-α (ng/mg prot)	18.99 ± 1.03^C^	17.77 ± 1.12^BC^	21.25 ± 1.03^AB^	22.93 ± 1.14^A^	0.15	<0.05	0.07

*RV−, infusing the essential medium; RV+, infusing the porcine rotavirus; CON, l-alanine-supplemented diet; Ile, l-isoleucine-supplemented diet*.

*^A,B,C^In the same row, values with different letter superscripts mean significant difference (*P* < 0.05)*.

Rotavirus infusion increased IFN-β (*P* < 0.05), IFN-γ (*P* < 0.05), TNF-α (*P* < 0.05), IL-1β (*P* = 0.08), and sIgA (*P* = 0.09) levels in the ileum of weaned piglets (Table 7). However, dietary l-isoleucine supplementation also enhanced the ileal IFNβ (*P* < 0.05), IFNγ (*P* < 0.05), IL-10 (*P* < 0.05), sIgA (*P* < 0.05), and IL-1β (*P* = 0.07) levels and further promoted IFNβ, IFNγ, IL-10 and sIgA concentrations in the ileum of weaned piglets infected by RV (*P* < 0.05, Table 7).

#### Gene Expressions of Cytokines in Ileum and Mesenteric Lymph Nodes

In the weaned piglets, RV infusion increased the mRNA expressions of IFNβ, IFNγ, IL-1β, and TNF-α in the ileum (*P* < 0.05, Table 8) and enhanced the mRNA expressions of IFNβ, IFNγ, IL-1β, IL-10, and TNF-α in the mesenteric lymph nodes (*P* < 0.05, Table 8). Supplementing l-isoleucine in diets also stimulated the mRNA expressions of IFN-β, IFN-γ, IL-10, IL-1β, and TNF-α in the ileum and mesenteric lymph nodes, although to different extents (*P* < 0.05 or *P* < 0.10, Table 8), and further enhanced the mRNA expressions of IFN-β, IFN-γ, IL-10, IL-1β, and/or TNF-α in the ileum and mesenteric lymph nodes of weaned piglets infected by RV (*P* < 0.05, Table 8).

**Table 8:** The effect of dietary l-isoleucine supplementation and/or rotavirus (RV) challenge on gene expressions of cytokines in the ileum and mesenteric lymph nodes of weaned pigs (*n* = 7).

	RV−		RV+		*P*-value		
	CON	Ile	CON	Ile	Ile	RV	Ile × RV
**Ileum**							
IFNβ	1.00 ± 0.26^C^	4.19 ± 0.26^AB^	3.41 ± 0.28^B^	5.17 ± 0.28^A^	<0.05	<0.05	0.07
IFNγ	1.00 ± 0.22^C^	1.55 ± 0.22^BC^	1.79 ± 0.22^B^	2.84 ± 0.22^A^	<0.05	<0.05	0.05
IL-1β	1.00 ± 0.39^B^	1.27 ± 0.48^B^	1.36 ± 0.48^B^	1.73 ± 0.56^A^	0.09	<0.05	0.17
IL-10	1.00 ± 0.32^BC^	2.05 ± 0.36^A^	0.42 ± 0.36^C^	2.61 ± 0.42^A^	<0.05	0.13	0.63
TNF-α	1.00 ± 0.19^B^	1.23 ± 0.18^B^	1.87 ± 0.21^A^	2.16 ± 0.17^A^	0.05	<0.05	0.34

**Mesenteric lymph nodes**							
IFNβ	1.00 ± 0.15^C^	1.39 ± 0.20^B^	1.37 ± 0.16^B^	1.76 ± 0.18^A^	<0.05	<0.05	0.07
IFNγ	1.00 ± 0.23^B^	1.48 ± 0.33^B^	1.52 ± 0.21^B^	2.42 ± 0.26^A^	<0.05	<0.05	0.09
IL-1β	1.00 ± 0.17^B^	1.24 ± 0.12^B^	1.49 ± 0.17^B^	2.84 ± 0.17^A^	<0.05	<0.05	0.14
IL-10	1.00 ± 0.11^C^	1.46 ± 0.21^B^	1.53 ± 0.19^B^	2.52 ± 0.17^A^	<0.05	<0.05	<0.05
TNF-α	1.00 ± 0.09^C^	1.43 ± 0.08^BC^	1.72 ± 0.10^B^	2.54 ± 0.12^A^	<0.05	<0.05	0.25

*RV−, infusing the essential medium; RV+, infusing the porcine rotavirus; CON, l-alanine-supplemented diet; Ile, l-isoleucine-supplemented diet*.

*^A,B,C^In the same row, values with different letter superscripts mean significant difference (*P* < 0.05)*.

#### Expressions of Genes Related to Innate Immune in Ileum and Mesenteric Lymph Nodes

In the weaned piglets, RV infusion, to different degrees, increased the mRNA expressions of NF-κB, TLR3, RIG-I, MDA5, TRIF, TRAF3, NEMO, TAK1, IRF3, MAVS, pBD2, and pBD3 in the ileum and/or mesenteric lymph nodes (*P* < 0.05 or *P* < 0.10, Table 9). In addition, l-isoleucine administration also enhanced the mRNA expressions of these genes related to innate immune in the ileum and/or mesenteric lymph nodes, although to different extents (*P* < 0.05 or *P* < 0.10, Table 9), and further enhanced the mRNA expressions of these genes related to innate immune in the ileum and/or mesenteric lymph nodes of weaned piglets infected by RV (*P* < 0.05, Table 9).

**Table 9:** The effect of dietary l-isoleucine supplementation and/or rotavirus (RV) challenge on expressions of genes related to innate immune in the ileum and mesenteric lymph nodes of weaned pigs (*n* = 7).

	RV−		RV+		*P*-value		
	CON	Ile	CON	Ile	Ile	RV	Ile × RV
**Ileum**							
NF-κB	1.00 ± 0.37^B^	1.75 ± 0.27^B^	1.21 ± 0.27^B^	2.84 ± 0.27^A^	<0.05	0.08	0.50
TLR3	1.00 ± 0.12^B^	1.51 ± 0.27^AB^	1.51 ± 0.29^B^	3.23 ± 0.27^A^	0.05	<0.05	0.59
RIG-I	1.00 ± 0.15^B^	2.20 ± 0.37^B^	2.26 ± 0.35^B^	5.07 ± 0.68^A^	<0.05	<0.05	0.09
MDA5	1.00 ± 0.26^C^	1.71 ± 0.44^BC^	1.96 ± 0.41^B^	3.37 ± 0.50^A^	0.06	<0.05	0.27
TRIF	1.00 ± 0.11^C^	1.31 ± 0.24^BC^	1.94 ± 0.21^B^	3.52 ± 0.20^A^	0.07	<0.05	0.13
TRAF3	1.00 ± 0.13^C^	1.65 ± 0.22^BC^	1.89 ± 0.31^B^	3.46 ± 0.32^A^	<0.05	<0.05	0.05
NEMO	1.00 ± 0.15^B^	1.56 ± 0.11^B^	0.84 ± 0.13^B^	2.34 ± 0.24^A^	<0.05	0.49	0.20
TAK1	1.00 ± 0.27^B^	1.74 ± 0.17^B^	1.21 ± 0.11^B^	2.93 ± 0.17^A^	<0.05	0.09	0.50
IRF3	1.00 ± 0.26^B^	2.07 ± 0.29^AB^	2.22 ± 0.26^AB^	2.71 ± 0.26^A^	<0.05	<0.05	0.32
MAVS	1.00 ± 0.21^C^	1.56 ± 0.21^BC^	1.70 ± 0.21^BC^	3.04 ± 0.24^A^	<0.05	<0.05	0.43
PG1–5	1.00 ± 0.17^B^	1.57 ± 0.12^B^	0.64 ± 0.13^C^	2.24 ± 0.24^A^	<0.05	0.49	0.20
pBD1	1.00 ± 0.57^B^	1.50 ± 0.80^B^	2.21 ± 0.99^AB^	3.35 ± 0.91^A^	0.08	<0.05	0.36
pBD2	1.00 ± 0.23^C^	3.62 ± 0.49^BC^	4.93 ± 0.49^B^	13.03 ± 0.39^A^	<0.05	<0.05	<0.05
pBD3	1.00 ± 0.23^C^	1.89 ± 0.26^B^	1.66 ± 0.56^B^	2.87 ± 0.26^A^	<0.05	0.08	0.78

**Mesenteric lymph nodes**							
NF-κB	1.00 ± 0.10^C^	1.23 ± 0.10^B^	1.21 ± 0.08^BC^	1.56 ± 0.10^A^	<0.05	0.09	0.66
TLR3	1.00 ± 0.15^C^	1.95 ± 0.20^B^	1.59 ± 0.14^BC^	4.46 ± 0.20^A^	<0.05	<0.05	0.05
RIG-I	1.00 ± 0.22^C^	1.48 ± 0.32^AB^	1.37 ± 0.32^B^	2.36 ± 0.32^A^	<0.05	<0.05	0.64
MDA5	1.00 ± 0.29^C^	1.22 ± 0.29^BC^	1.70 ± 0.28^B^	2.36 ± 0.29^A^	0.08	<0.05	0.27
TRIF	1.00 ± 0.19^B^	1.54 ± 0.27^B^	1.55 ± 0.17^B^	3.42 ± 0.18^A^	<0.05	0.07	0.50
TRAF3	1.00 ± 0.21^C^	1.48 ± 0.32^AB^	1.37 ± 0.32^B^	2.36 ± 0.32^A^	<0.05	<0.05	0.64
NEMO	1.00 ± 0.13^B^	1.23 ± 0.10^B^	1.21 ± 0.08^B^	1.56 ± 0.10^A^	<0.05	0.08	0.66
TAK1	1.00 ± 0.19^C^	1.55 ± 0.31^BC^	1.71 ± 0.22^BC^	3.14 ± 0.23^A^	<0.05	<0.05	0.43
IRF3	1.00 ± 0.19^C^	1.53 ± 0.28^B^	1.46 ± 0.19^B^	3.01 ± 0.19^A^	<0.05	<0.05	0.50
MAVS	1.00 ± 0.17^C^	1.96 ± 0.17^B^	1.46 ± 0.25^BC^	2.48 ± 0.25^A^	<0.05	<0.05	0.61

*RV−, infusing the essential medium; RV+, infusing the porcine rotavirus; CON, l-alanine-supplemented diet; Ile, l-isoleucine-supplemented diet*.

*^A,B,C^In the same row, values with different letter superscripts mean significant difference (*P* < 0.05)*.

## Discussion

As a function amino acid, isoleucine was well known as its effects on glucolipid metabolism and protein synthesis. But recently studies have shown that supplementing l-isoleucine can improve the health status and growth of human infants and young animals that were challenged by some pathogens (such as *Escherichia coli*) *via* improving the immune function (10, 14, 18, 19). l-Isoleucine treatment should improve the innate immunity possibly *via* some signaling pathways, such as TLRs, RIG-I, MAVS and MDA5, which can stimulate the generation and expression of anti-pathogen components. In this study, l-isoleucine treatment significantly stimulated the expressions of TLR3, RIG-I, MDA5, MAVS, IRF3, NF-κB, IFNβ, IFNγ, pBD2, and/or pBD3 in IPEC-J2 and 3D4/31 cells. These genes play the important role for preventing invasion of dsRNA viruses (including rotavirus). Therefore, l-isoleucine administration could alleviate the effect of rotavirus on humans and animals.

Rotavirus has been considered as a major pathogen causing severe gastroenteritis in infants and young children, as well as in other young animals (3, 32–35). Many previous study have reported that RV infection resulted in growth performance reduction, inflammation response, severe diarrhea, changes of cytokines and generation of RV-ab in piglets (15, 26, 29, 35–38), which were similar with this study. However, there was a few difference of RV infection affecting cytokines’ levels in serum and intestine between this study and our previous studies, which could be related to experimental duration and RV-infused dose. Besides, this study observed that RV infection enhanced the mRNA expression of genes related to innate immune in the ileum and/or mesenteric lymph nodes, increased some free amino acid concentrations in serum, and decreased the quantity of CD4^+^ and CD4^+^/CD8^+^ ratio in peripheral blood and the concentration of NSP4 in ileal mucosa. Therefore, it is proposed that RV infection model was successfully established.

Previous studied indicated that dietary l-isoleucine supplementation could promote growth of piglets, fattening pigs, laying hens and juvenile Jian carp (39–43). In this study, l-isoleucine administration could improve ADFI, ADG and F/G ratio of piglets before RV infusion and also enhanced ADFI and G under the condition of RV infection. In addition, dietary l-isoleucine supplementation could increase serum isoleucine and arginine levels, and decrease serum UN and glycine concentrations in the normal piglets, then assuaged the increasing serum UN and glycine concentrations in RV-infected piglets. Serum UN and free amino acid concentration can directly reflect the level of amino acid utilization and protein metabolism in the whole body (44, 45). These indicated that RV infection impaired protein metabolism, which could be alleviated by l-isoleucine administration. Therefore, dietary l-isoleucine supplementation enhanced the growth performance of the RV-infected piglets potentially through improvement of protein metabolism and utilization in whole body.

Immune function, including the humoral and cellular immune, is critical to obliterate the pathogens, which could keep the health and growth of animals and human. Various immunoglobulins (such as IgA, IgG and some specific antibodies) are the main constituents of humoral immune (46), in which sIgA is important to protect the gut mucosa from the pathogen invasion (47). In this study, we observe that supplementing l-isoleucine in diets increased serum IgA, IgG and RV-ab levels, and ileal sIgA and RV-ab concentrations in weaned piglets under normal and/or RV-infected conditions, which is similar to the results in different animals (15, 48, 49). It was possible that l-isoleucine elevate humoral immune of weaned piglets infected by RV *via* upregulating the immunoglobulin generation.

In this study, we also found that l-isoleucine treatment significantly attenuated the decrease of CD4^+^ T cells in the peripheral blood of RV-infected piglets. As a part of cellular immune, CD4^+^ T cells contribute a myriad of activities for protecting animals and human against the pathogens (50). Especially, CD4^+^ T cells are essential as helper to enhance antibody production and response in B cell (51). Thus, these could partially explain the effect of dietary l-isoleucine supplementation on generation of immunoglobulins and RV-ab.

Cytokines are the key in keeping cell-to-cell communication, and regulate lymphocyte differentiation and maturity, and subsequent functional activity of the peripheral immune compartment (52, 53). The cytokines balance (especially Th1/Th2) and levels are critical to immunity maintenance and inflammation control. Moreover, type I IFN and pro-inflammation cytokines can effectively prevent the invasion and replication of virus, including RV (5, 6). The previous studies have shown that l-isoleucine administration affects the concentration of IL-1β, IFNβ and IFNγ in serum and ileum of weaned piglets (18) and regulates IL-1β, IL-4, IFNβ, and IFNγ generation in human peripheral blood mononuclear cells (18, 54). This study also revealed that dietary l-isoleucine supplementation elevated serum and ileal IL-1β, IFNβ, IFNγ, TNF-α, and IL-10 levels and further promoted serum and ileal concentrations of these cytokines in RV-infected piglets. Although the different lymphocyte subpopulation can affect antiviral capacity *via* regulating the production and secretion of cytokines (50), l-isoleucine treatment increasing cytokines’ levels, especially in the ileum, could not completely be explained by the improvement of CD4^+^ T cells. Meanwhile, in the normal and RV-infected piglets, the mRNA expressions of IL-1β, IFNβ, IFN-γ, TNF-α, and IL-10 were also upregulated by l-isoleucine administration in the ileum and mesenteric lymph nodes that are targets of RV challenge.

Innate immunity is the first and universal defense mechanism of host against various pathogens such as bacteria and viruses (55). Defensins, as a kind of antimicrobial peptides produced by leukocytes and epithelial cells, play an important role in antiviral strategy *via* acting directly on virion and host cells (56). In accordance with many studies, we also found that l-isoleucine treatment enhanced the expression of pBD2 and/or pBD3 in the *in vitro* and *in vivo* experiments (10, 11). It is possible that l-isoleucine preventing the invasion of RV be relative with the increase of pBD2 and pBD3 production.

Host–pathogen interactions are generally started *via* host recognition of pathogen-associated molecular patterns (PAMPs) that are essential for the survival of pathogens (i.e., dsRNA of RV) (55, 57, 58). And these PAMPs are sensed by the host’s PRRs, including toll-like receptors, RIG-I-like receptors, NOD-like receptors and DNA receptors, which are expressed on innate immune cells (59, 60). Effective sensing of PAMPs rapidly induces host immune response *via* activating several signaling pathways (such as IRFs, NF-κB, and MAVS) that lead to the transcription and release of various cytokines, type I IFN and defensins, which subsequently enhances the eradication of pathogens (61). Moreover, among these PRRs, TLR3, RIG-I, and MDA5 have the property of recognizing the dsRNA-virus (i.e., RV) (6, 62).

Several previous studies determined that, following infection, RV could interact with IRF3 and NF-κB, and result in the proteasome-mediated degradation of IRF3 and NF-κB, and then suppress host’s normal immune antiviral immune response and cytokines secretion, which is one of the reasons that RV lead to a severe gastroenteritis (63, 64). This study also showed that dietary l-isoleucine supplementation enhanced the mRNA expressions of NF-κB, TLR3, RIG-I, MDA5, TRIF, TRAF3, NEMO, TAK1, IRF3, and MAVS in the ileum and/or mesenteric lymph nodes of normal and RV-infected piglets, which is consistent with the results of *in vitro* experiment. l-Isoleucine has the property of stimulating PRRs and the relative pathways, which will promote the transcription and generation of antiviral proteins, including cytokines and defensins. Therefore, supplementing l-isoleucine in diets relieving the diarrhea induced by RV challenge could be due to the activation of innate immunity before infection. Previous study indicated that NSP4 is the main cause of piglet diarrhea, and it is also a good marker of RV level in tissue (65). This study showed that dietary l-isoleucine supplementation decreases the NSP4 level in ileum of infected piglet. Thus, it is possible that dietary l-isoleucine supplementation decreased RV level in intestine through improving the immune function of infected piglets.

Previous study indicated that oral l-isoleucine solution tended to relieve the cute diarrhea induced by mixed pathogens (including RV) in children (19), and it is similar with our findings. This study can also explain how l-isoleucine relieved the diarrhea and infection induced by RV. In addition, l-isoleucine administration can also relieve *E. coli* infection and its impairment (41), but the recognition of *E. coli* was not associated with TLR3, RIG-I, and MDA5. Thus, the effect of isoleucine on other PRRs expression needs to be further studied.

In summary, RV infection impaired growth performance and immune response in weaned piglets. However, l-isoleucine supplementation relieved the negative effect of RV infection on growth performance and diarrhea of piglets, which could be due that l-isoleucine treatment improved the immune response, especially generation and secretion of antiviral proteins, *via* activating PRRs and the relative pathways. However, the further mechanisms of isoleucine regulating immunity also need to be studied in the future. The gastrointestinal and immune systems of resemble those of human infants, so piglets are an ideal animal model on studying RV infection and immune response. Thus, according to this study, we can propose that l-isoleucine may be also used in the prevention and therapy of infant RV infection.

## Ethics Statement

This study was carried out in accordance with the recommendations of Animal Welfare Regulations, Animal Care Advisory Committee of Sichuan Agricultural University. The protocol was approved by the Animal Care Advisory Committee of Sichuan Agricultural University.

## Author Contributions

Conceived and designed the experiments: DC, BY, JH, XM, GT, and MR. Performed the experiments: CG, QY, XM, and JY. Analyzed the data: JW, PZ, and JL. Contributed reagents/materials/analysis tools: JW and MR. Wrote the paper: CG and XM.

## Conflict of Interest Statement

The authors declare that the research was conducted in the absence of any commercial or financial relationships that could be construed as a potential conflict of interest.
