# Supplementary material for: l-Isoleucine Administration Alleviates Rotavirus Infection and Immune Response in the Weaned Piglet Model
Source: Front Immunol. 2018 Jul 16;9:1654. doi: 10.3389/fimmu.2018.01654 (PMC6054962; doi:10.3389/fimmu.2018.01654)
Supplement: Supplementary file 1 [file Table_1.docx]

Table S1. Primers and annealing temperatures used in real-time quantitative PCR

| Primer names | Sequence (5′–3′) | GenBank ID |
| --- | --- | --- |
| TLR3-F | CTCGCTGATTCTCCTCTTCTC | NM_001097444 |
| TLR3-R | TCTCCATTCCTGTCCTGTGA |  |
| RIG-I-F | CACACCAAGAGCCCAAAC | EU126659 |
| RIG-I-R | TGACCCGATAGCAACAGC |  |
| MDA5-F | CCGTTTACCGATCCGACAGA | EU006039 |
| MDA5-R | GCAAATCAGCTGGTGCATTGA |  |
| MAVS-F | AGAAGCAGGACACAGAAC | JN644269 |
| MAVS-R | GAAGGAGACAGTCGGAGA |  |
| TRIF-F | CAAGTGGAGGAAGGAACAGG | XM_003362039.1 |
| TRIF-R | CAACTGCGTCTGGTAGGACA |  |
| TRAF3-F | AGGAGAAGTTTGTGAAGACGGTGGA | NC_000014.9 |
| TRAF3-R | TACCTCAGTTCGTTTTTCTACCTCA |  |
| TAK1-F | GGTTTGCCAGAATCGGAGT | NC_004354. |
| TAK1-R | TGTAAATACTCCTCTGTGCCGT |  |
| NEMO-F | AGATGCTGAGGGAACGCTGTGAGG | NC_000017.11 |
| NEMO-R | TACTCGTCCGTGGAGACCTTCTTAG |  |
| NF-κB-F | CATCTTTGACAACCGTGCCC | NM_001114281.1 |
| NF-κB-R | GGGCCCGTGAAATACACCTC |  |
| IRF3-F | TTTAGCAGAGGACCACAGCA | NM_213770 |
| IRF3-R | CCCACTCGTCGTCATTCG |  |
| IFNβ-F | CGATACCAACAAAGGAGCAG | GQ415073 |
| IFNβ-R | GGTTTCATTCCAGCCAGT |  |
| IFNγ-F | TGGTAGCTTCTGGGAACTGAATG | NM_213948 |
| IFNγ-R | AGGCTTTCGCGCTGGATCTGC |  |
| IL-10-F | TGCTCTATTGCCTGATCTTCCTG | JQ687536 |
| IL-10-R | CCCATCTGGTCCTTCGTTTG |  |
| IL-1β-F | ACCTGGACCTTGGTTCTC | NM_214055 |
| IL-1β-R | GGATTCTTCATCGGCTTC |  |
| TNF-α-F | ACGCTCTTCTGCCTACTGC | JF831365 |
| TNF-α-R | TCCCTCGGCTTTGACATT |  |
| β-actin-F | TCTGGCACCACACCTTCT | AJ312193 |
| β-actin-R | TGATCTGGGTCATCTTCTCAC |  |
| pBD-2-F | TGTCTGCCTCCTCTCTTCC | AY506573 |
| pBD-2-R | AACAGGTCCCTTCAATCCTG |  |
| pBD-3-F | CCTTCTCTTTGCCTTGCTCTT | AY460575 |
| pBD-3-R | GCCACTCACAGAACAGCTACC |  |
